# Dietary Fiber Intake and Metabolic Syndrome Risk Factors among Young South African Adults

**DOI:** 10.3390/nu10040504

**Published:** 2018-04-18

**Authors:** Machoene D. Sekgala, Zandile J. Mchiza, Whadi-ah Parker, Kotsedi D. Monyeki

**Affiliations:** 1 Population Health, Health Systems and Innovations, Human Science Research Council, Cape Town 8001, South Africa; wparker@hsrc.ac.za; 2 Department of Physiology and Environmental Health, University of Limpopo, Polokwane 0727, South Africa; kotsedi.monyeki@ul.ac.za; 3 Faculty of Community and Health Sciences, School of Public Health, University of the Western Cape, Bellville 7535, South Africa; jmchiza@uwc.ac.za or 1zandilem@gmail.com

**Keywords:** fiber intake, metabolic syndrome, rural population, blood pressure

## Abstract

This study attempts to bridge the research gap regarding the importance of dietary fiber in reducing metabolic syndrome (MetS) risk factors in young rural South Africans. A total of 627 individuals (309 males and 318 females) aged 18–30 years participated in the study. Dietary intake was measured using a validated 24-h recall method. The consumption of different types of dietary fiber (total, soluble, and insoluble) was calculated and presented as grams. Anthropometrics, blood pressure, fasting blood glucose, and lipid profiles were measured according to standard protocols. According to the definition of the International Diabetes Federation (IDF), the prevalence of MetS was 23.1%. Overall, the total median [interquartile range (IQR)] values for total, insoluble, and soluble fiber consumed were 4.6 g [0.0–48.9], 0.0 g [0.0–18.0], and 0.0 g [0.0–15.0], respectively. Females had a higher median [IQR] intake of total (5.1 g [0.0–48.9] vs. 4.3 g [0.0–43.9]), insoluble (0.0 g [0.0–18.0] vs. 0.0 g [0.0–12.0]), and soluble fiber (0.0 g [0.0–14.9] vs. 0.0 g [0.0–7.3]) than males, respectively. The mean values for waist circumference, fasting blood glucose, and total cholesterol were higher in females than males (82.20 cm vs. 75.07 cm; 5.59 mmol/L vs. 5.44 mmol/L; and 4.26 mmol/L vs. 4.03 mmol/L, respectively), with significant differences observed for waist circumference and total cholesterol (*p* < 0.001 and *p* = 0.005, respectively). More than 97% of participants had fiber intakes below the recommended levels. After adjusting for all potential confounders (age, gender, and energy), log total fiber was inversely associated with fasting blood glucose (β = −0.019, 95% CI [−0.042 to 0.003], *p* < 0.05), systolic blood pressure (β = −0.002, 95% CI [−0.050 to 0.002], *p* < 0.05) and high-density lipoprotein cholesterol (β = −0.085, 95% CI [−0.173 to 0.002], *p* = 0.051) This study may be of public health relevance, providing a potential link between less dietary fiber intake and fasting blood glucose (FBG) and both systolic and diastolic blood pressure. Therefore, this observational data encourages public health policy measures to increase the consumption of dietary fiber in rural communities in order to lower the burden of MetS and its associated risk factors.

## 1. Introduction

Current and available national and regional South African studies, suggest that the dietary fiber consumption among South Africans 15 years and older does not meet the recommended daily intake [1,2,3,4]. Certain segments of the population are more affected than others, these include the poorer, less urbanized (peri-urban, townships) and rural communities. Similarly, current estimates and projections suggest that the burden of non-communicable diseases (NCDs) in South Africa is unique and is rapidly growing in all communities, including rural and poorer communities [5,6,7,8,9,10]. Metabolic syndrome (MetS) has been considered the fastest developing NCD syndrome in the world [11,12]. Based on the available South African studies, the prevalence of metabolic syndrome varies between provinces and ranges from 42.6% to 62.0% in the Western Cape [6,13], 5.9% in the Eastern Cape [14], 22.1% in KwaZulu-Natal [15], and 52.2% and 39.7% in rural and urban areas of the Free State, respectively [9]. Additionally, one African study reported that, dietary fiber is associated with a reduced likelihood of having MetS [16]. On the other hand, the majority of these studies reported the prevalence of specific MetS risk factors instead of the prevalence of MetS. To mention a few, the data from the first South African National Health Examination Survey (SANHANES-1) indicated that 35.1% of South Africans had hypertension [17], and 10.1% of South Africans had diabetes [18].

Substantiated evidence has also shown a rapid rise of NCDs in Africa, especially in South Africa [19]. This can be attributed to the rapid nutrition transition associated with urbanization, including the adoption of “Westernized” diets—diets high in refined carbohydrates, low in fruit and vegetables, and deficient of fiber—amongst others [19,20]. In fact, Steyn and Mchiza [20] have shown that in recent years, South Africa has been identified as one of the African countries with an abundance of refined sugar; however, it has also been identified as one of the countries that has the lowest availability of fruit and vegetables. Low fruit and vegetable consumption is further exacerbated by economic constraints, cultural diversity, and ethnic food preferences [1,21]. In some poorer and black ethnic communities in the country, high fiber food, including fruit and vegetables, consumption is reduced when foods that are rich in fat and refined sugars become available. This has been attributed to the belief that these communities have missed the opportunities of enjoying these foods due to previous deprivation [22]. Furthermore, Mchiza et al. [23] has shown that only 26% of the food sold in the streets of South Africa are fruit and vegetables, while Hill et al. [24] have shown that only 27% and 5% of South African consumers prefer purchasing fruit and vegetables daily, respectively.

This is a cause for concern given that low fiber intake through fruit and vegetables is implicated in the mortality and the burden of disease associated with ischemic heart and stroke diseases, as well as colorectal, esophageal, lung, and gastric cancers in the country [25]. The aforementioned South African evidence is also corroborated by international evidence that implicates low dietary fiber intake in the etiology of NCDs through different biological mechanisms [26]. Some of these studies suggest that the daily consumption of the recommended amounts of fiber is beneficial in that it expedites the movement of waste products through the intestinal tract, thereby decreasing the gut transit time and protecting the gut from harmful waste that may support the development of different forms of gut cancers [25].

The dietary guidelines of the American Heart Association [27] and the Food and Nutrition Board (FNB) [28] emphasize a dietary pattern that is high in fruit and vegetables (5 to 9 servings per day) and an increased fiber intake to about 25 g for adult females and 38 g for adult males (of which 2.5 g to 10.3 g should be soluble fiber) as an effective intervention to decrease the development of metabolic risk factors [29,30]. Dietary fiber is known to have prebiotic properties that promote the growth of good gut flora [31]. Furthermore, dietary fiber, including soluble fiber, also reduces the glycemic response leading to lower insulin stimulation of hepatic cholesterol synthesis [32,33,34]. It is important to note that most food sources of fiber (especially fruit and vegetables) are also good sources of flavonoids, which are known to act as antioxidants [35]. Flavonoids have also been shown to have antidiabetic properties and reduce the risk of cardiovascular disease through the inhibition of the oxidation of low-density lipoprotein (LDL)-cholesterol (LDL-C) and reducing both platelet aggregation and ischemic damage [36,37]. To our knowledge, there are no primary or original studies that have investigated the role of fiber on the development of MetS risk factors in South Africa. However, in 2000, Schneider et al. [38] modeled data from different studies in South Africa to estimate the burden of disease attributable to low fruit and vegetable intake (below the recommended 5 servings (<400 g/day)) in South Africa. Their findings suggested that the low intake of fruit and vegetables accounted for 3.2% of total deaths and 1.1% of the 16.2 million deaths attributable to mortality and disability-adjusted life years. Schneider et al. [38] concluded that their results support the finding that a high fruit and vegetable intake contributes significantly to decreasing mortality from certain diseases and therefore recommended an increase in fruit and vegetables in the diet of South Africans.

Internationally, studies have implicated age and gender in the etiology of MetS and its associated risk factors [15,39,40,41,42]. For decades, MetS was recognized as a disorder of the elderly, and as such, its definition was based on adult health [43,44,45,46]. In the last two decades, several international studies have shown that MetS and its risk factors are evident in younger age groups, including children [40,41,42,47]. Berenson et al. [48] have also long shown a consistent trend toward a greater prevalence of coronary-artery lesions with an increase in age. Although, there have been studies looking at various components of MetS in selected age groupings, no studies have looked solely at dietary fiber intake between different age groups especially in a rural population. We lack information on dietary fiber intake among the participants across the age groups. Hence, the current study presented the data in two different age groups. Furthermore, the majority of studies reported that females are more likely to develop MetS than males especially in developed and high socio-economic status areas [15,41,42,47,49]. Little is known about the influence of dietary fiber intake on MetS among rural populations.

The current study therefore attempts to bridge the abovementioned research gap by producing original and primary data regarding the importance of dietary fiber in reducing MetS risk factors in rural communities of South Africa. The study uses data from a least-served and poorer community in the Limpopo Province of South Africa (Ellisras) and quantifies the consumption of dietary fiber among males and females aged 18–30 years. Given that previous studies have shown distinct differences in MetS between age groups and between genders, the current study has been designed to reflect the differences in both gender and age (albeit a narrow age range). It also reports the types of dietary fiber (resistant [soluble/insoluble]) consumed and the relationship between different types of fiber and the MetS risk factors. This information has policy implications because it identifies a window of opportunity for intervention and offers a practical, natural, and affordable alternative to prevent and curb the escalating prevalence of MetS in rural and least-served communities in the country.

## 2. Materials and Methods

### 2.1. Geographical Area

Ellisras (known as Lephalale) is considered to be one of the deep rural areas in the western part of the Limpopo Province in South Africa. The villages are approximately 70 km away from the Ellisras town (23°40 S 27°44 W), adjacent to the Botswana border. The population is about 50,000, dispersed across 42 settlements [50]. The main sources of employment for the Ellisras residents are the Iscor coal mine and the Matimba electricity power station. The remaining workforce is mostly involved in subsistence farming and cattle rearing, while a few are involved in education and civil services. Poverty, unemployment, and low life expectancy is common part in rural South African settings, and the Ellisras rural population is no exception [51].

### 2.2. Sample and Research Design

The detailed sample size selection for the current study is presented elsewhere [52,53]. In summary, the current study forms part of the Ellisras Longitudinal Study (ELS), which followed a cluster sampling method. The selection was carried out randomly from 68 government-subsidized schools within the Ellisras area. The initial sample was collected from 22 schools (10 preschools and 12 primary schools) in 1996 and followed over 20 years. The current sample therefore included a total of 627 individuals (309 males and 318 females) who were participants in the ELS and who were within the ages of 18–30 years in 2015. The Ethics Committee of the University of Limpopo granted ethical approval prior to the survey with ethical clearance number MREC /P/204/2013: IR, and the participants signed informed consent forms.

### 2.3. Data Collection

#### 2.3.1. Dietary Intake

Dietary intake was measured using a validated 24-h recall method [54]. Senior, Northern Sotho-speaking, dietetic students of the University of Limpopo, specifically trained to use the 24-h recall method, completed interviews with the participants regarding their dietary intake over the previous 24 h. For each participant, interviews took place on one weekday and on one weekend day. An average of the two days of 24-h dietary intake was then made for each participant. Estimated portion sizes of foods consumed were recorded in as much detail as possible, using a pretested questionnaire and food models simulating average portions of local foods [55]. The different forms of dietary fiber (total, soluble, and insoluble fiber) consumed were calculated using the Food Finder 111 analysis package, recorded, and presented as grams in the current manuscript. Briefly, the amount of food items consumed by each individual (breakfast, lunch, supper) was captured into the Food Finder 111 software version 3.0 (May 2014) [56]. All food items were analyzed, and the output was saved on to an excel spreadsheet. The raw data was imported into the statistical package of the social sciences (SPSS) version 24.0 for statistical analysis.

#### 2.3.2. Anthropometric and Blood Pressure Measurements

Anthropometric measurements (waist circumferences) were conducted on all the study participants, according to the standard procedures of the International Society for the Advancement of Kinanthropometry [57]. The waist circumference measurements were taken to the nearest 0.1 cm using a soft measuring tape.

Using an electronic Micronta monitoring kit, three blood pressure (BP) readings were taken after the subject had been seated for 5 min or longer. The bladder of the device contains an electronic infrasonic transducer that monitors BP and pulse rate, displaying these concurrently on the screen. This versatile instrument has been designed for research and clinical purposes [58]. Anthropometric and blood pressure measurements were completed by an ELS primary investigator with help from medical students from Vrije University in Amsterdam and ELS administrators.

#### 2.3.3. Biochemical Parameters

Fasting venous blood specimens were collected from all the participants for the measurement of blood glucose, total cholesterol, triglycerides, and high-density lipoprotein (HDL)-cholesterol (HDL-C). Blood specimens for the measurement of fasting venous plasma glucose (FVPG) were drawn into fluoride tubes. The FVPG was measured using the glucose oxidase method, on a Beckman LX20^®^ auto-analyzer (Beckman Coulter, Fullerton, CA, USA) after the samples were centrifuged within 4 h. The enzymatic assay kits on a Beckman LX20^®^ auto-analyzer (Beckman Coulter, Fullerton, CA, USA) were used to measure the serum lipid profile. The blood samples were collected by qualified nurses from Witpoort Hospital in Ellisras and analyzed by University of Limpopo Medical and Pathology laboratory staff.

MetS was diagnosed using the new harmonized guidelines of the international diabetes federation (IDF), which requires a waist circumference (WC) of ≥94 cm (Males) and ≥80 cm (Females) in addition to two of the following criteria: low high-density lipoprotein cholesterol (HDL-C) (<1.0 mmol/L Male; <1.3 mmol/L Female), high triglycerides (≥1.7 mmol/L), elevated blood pressure (≥130 mmHg systole and/or ≥85 mmHg diastole), or fasting blood glucose (FBG) (≥5.6 mmol/L) [59].

### 2.4. Statistical Analysis

Descriptive statistics were used to describe the participants’ characteristics. Chi-squared tests were used for proportions between genders. Linear regression was used to determine the relationship between fiber intake and the MetS risk factors, both unadjusted and adjusted according to age, gender, and total energy. Dietary fiber variables (total, insoluble, and soluble fiber) used in linear regression method were log transformed prior to analysis, because they had skewed distribution. The SPSS version 24.0 was used for data analysis. A *p*-value of <0.05 and confidence intervals that did not overlap indicated statistical significance. Data is presented in percentages, means, medians, interquartile ranges, and standard deviation values. Dietary fiber was calculated using South African food analysis software (Food Finder 111 software version 3.0 (May 2014)) [56], and the outcomes obtained were compared to the recommended dietary fiber intake [60].

## 3. Results

Overall, 627 young adults residing in Ellisras participated in the current study. Of the total, 38% of the participants were aged 18–24 years, and 62% were aged 25–30 years. There was an almost equal spread between males and females (309 [49.2%] and 318 [50.7%], respectively). The participants were from a rural and financially constrained community. The prevalence of MetS in rural young adults was 23.1% (8.6% of males and 36.8% of females).

Table 1 shows the standard deviation, median values, and interquartile ranges for fiber consumption among the participants. Overall, the total median [IQR] values for total, insoluble, and soluble fiber consumed were 4.6 g [0.0–48.9], 0.0 g [0.0–18.0] and 0.0 g [0.0–15.0], respectively. Females had a higher median [IQR] intake of total (5.1 g [0.0–48.9] vs. 4.3 g [0.0–43.9]), insoluble (0.0 g [0.0–18.0] vs. 0.0 g [0.0–12.0]), and soluble fiber (0.0 g [0.0–14.9] vs. 0.0 g [0.0–7.3]) than males, respectively. Younger participants (18–24 years) consumed less, total (5.3 g [0.0–32.7] vs. 4.0 g [0.0–48.9]), insoluble (0.0 g [0.0–10.9] vs. 0.0 g [0.0–18.0]), and soluble fiber (0.0 g [0.0–12.9] vs. 0.0 g [0.0–14.9]) as compare with the older (25–30 years) participants, respectively).

Table 2 shows the mean values for the MetS risk factors. Overall, the mean values for waist circumference, fasting blood glucose, and total cholesterol were higher in females than in males (82.20 cm vs. 75.07 cm; 5.59 mmol/L vs. 5.44 mmol/L; and 4.26 mmol/L vs. 4.03 mmol/L, respectively), with significant differences observed for waist circumference and total cholesterol (*p* < 0.001 and *p* = 0.005, respectively). Similar significant gender differences were observed in the 25–30-year-old participants; however, in the younger group (18–24-year-old participants), the differences remained only for waist circumference. In terms of HDL-cholesterol, triglycerides, and systolic and diastolic blood pressure, the mean values were significantly higher in males compared with those in females (1.2 mmol/L vs. 1.1 mmol/L, *p* < 0.001; 1.1 mmol/L vs. 0.96 mmol/L, *p* = 0.035; 125.9 mmHg vs. 114.1 mmHg, *p* < 0.001; and 71.4 mmHg vs. 69.1 mmHg, *p* = 0.003, respectively). These gender differences were also observed within the younger and older age groups but were significant for HDL-cholesterol and systolic and diastolic blood pressure in the 25–30-year-old participants and only for HDL-cholesterol and systolic blood pressure in the 18–24-year old participants.

Table 3 shows the prevalence of MetS, as well as the consumption of dietary fiber intake and MetS risk factors, by age group and gender. The overall prevalence of MetS was 23.1%, with significantly more females than males presenting with MetS (36.8% vs. 8.6%, respectively). Similar results were observed in both age groups, where more females than males presented with MetS in the 18–24-year-old group (30.8% vs. 9.8%, respectively) and in the 25–30 years old group (40.3% vs. 7.7%, respectively). Overall, the majority (97.6%, 99.5%, and 99.7%) of the participants had total, insoluble, and soluble fiber intakes below the recommended levels (<25 g for females and <38 g for males). Significant gender differences were shown for total fiber consumption only, where significantly more females than males consumed fiber levels that were below the daily requirements (99.4% vs. 96.4%, *p* = 0.008). Significantly more females had larger waist circumferences (52.6% vs. 4.6% *p* < 0.001), higher total cholesterol (22.3% vs. 11.2%, *p* < 0.001), and lower HDL-cholesterol (78.6% vs. 29.9%, *p* < 0.001) than males. Significant gender differences were observed in both younger (18–24 years) and older (25–30 years) age groups for waist circumference and HDL-cholesterol. In addition, significant differences in gender for total cholesterol were only observed in the older (25–30 years) age group. Regarding blood pressure, significantly more males than females presented with elevated systolic (39.2% vs. 7.7%, *p* < 0.001) and diastolic blood pressure (8.9% vs. 5.0%, *p* = 0.52 [tending to significance]). Significant gender differences were observed in both young and older age groups for systolic blood pressure, while differences in diastolic blood pressure were only observed in the older age group.

Table 4 shows the linear regression analysis undertaken to show the association between log dietary fiber consumption and different metabolic syndrome risk factors. Low and negative significant (*p* < 0.05) associations between log total dietary fiber and fasting blood glucose and systolic blood pressure were observed. After adjusting for all potential confounders (age, gender, and energy), log total fiber was inversely associated with high-density lipoprotein cholesterol. Log insoluble fiber was inversely associated with systolic blood pressure only after adjusting for age and gender. Finally, log soluble fiber was inversely associated with systolic blood pressure.

## 4. Discussion

The total dietary fiber intake by the majority of participants in the current study is far lower than the dietary fiber reference intakes of 25 g for females and 38 g for males, and the most affected groups are the older adults (25–30 years) and males. This is a cause for concern given that sources of fiber (cereals and grains, as well as fruit and vegetables) are assumed to be abundant in the villages in Limpopo where the participants resided, and residents in these villages rely on subsistence farming as their source of food production. However, it is important to emphasize that low levels of dietary fiber intake have been reported previously in other South African studies also undertaken in rural and peri-urban communities in South Africa [2,3,4,20,61]. In fact, Richter et al. [62] have shown that fiber intake is low in adults residing in rural areas in the Northwest provinces of South Africa where Limpopo is situated. This is a cause for concern given the increased benefits that people are likely to experience if they consumed this nutrient. Substantiated evidence suggests that high dietary fiber intake lowers adiposity since it suppresses appetite [63]. Moreover, fiber is beneficial in that it expedites the movements of waste products through the intestinal tract, thereby decreasing the gut transit time to protect the gut from harmful waste that may support the development of different forms of gut cancers [64]. Moreover, dietary fiber is important in that is has antidiabetic and antihypertensive properties, inhibits the oxidation of LDL-cholesterol, reduce platelet aggregation, and later reduces ischemic damage [65].

Other notable results in the current study were that females tended to consume more dietary fiber compared with males, while 18–24-year-old participants consumed less dietary fiber that the 25–30-year-old participants. While the results are corroborated by some South African studies [2,66] in terms of age and fiber consumption, they contrast with other South African evidence [3,4,20,61] that suggests that males have a higher dietary fiber intake than females. It is also important to note though, that despite females in the current study consuming significantly higher levels of dietary fiber than men, a higher proportion of them consumed lower levels of total dietary fiber than the recommended allowances when compared with the proportion of men.

International studies suggest that dietary fiber intake is a predictor of MetS risk factors [27,28,29,30,67]. While it may appear as though the mean MetS risk factor values presented in the current study are within the normal range, as in other South African studies, the majority of females participating in the current study had larger waist circumferences, higher total cholesterol, and lower HDL-cholesterol than the recommended allowances when compared with their male counterparts [15,68]. Most males, on the other hand, have higher systolic and diastolic blood pressure when compared with their female counterparts. It is important to note that a sizable proportion of the participants in the current study presented with abnormal values for the MetS risk factors. These participants also consumed dietary fiber that is lower than the recommended levels. As such, they have not allowed themselves to benefit from the known effect of high fiber consumption on preventing the development of the MetS risk factors.

Most of the MetS risk factors in the current study did not show significant association with the different forms of log dietary fiber consumed except for systolic blood pressure (SBP), diastolic blood pressure (DBP), FBG and HDL-C. The lack of association of log dietary fiber with these MetS risk factors in the current research could be explained by the fact that dietary fiber is effective only when higher levels are consumed. In fact, soluble fiber has been identified as the type of fiber that provides the most significant effect for most MetS risk factors [30]. The majority of participants in the current study reported a negligible/extremely low intake of soluble fiber, hardly reaching 2 g per day. Hence, it may appear to have had no significant effect on the waist circumference and total cholesterol and triglycerides levels of these participants. In their review, Brown et al. [30] emphasized that soluble dietary fiber of about 2–10 g per day has a small but significant effect on the total cholesterol of individuals. Brown et al. [30] and the Food and Drug Administration [69] suggest that studies executed internationally emphasize that the effectiveness of fiber is dose-dependent in that doses ranging from 34 g total dietary fiber (2.5 g soluble fiber) to 123 g (10.3 g soluble fiber) can decrease total cholesterol by 20.045 mmol/L (95% CI: 20.054, 20.035) or higher, while they decrease LDL-cholesterol by 20.057 mmol/L (95% CI: 20.070, 20.044) or higher.

Finally, Ahola et al. [67] have shown that a high consumption of complex carbohydrates, fruit and vegetables, and dietary fiber tends to decrease the likelihood of individuals presenting with abnormal adiposity, serum cholesterol, and both systolic and diastolic blood pressure, and this association is shown to be dependent on gender. As noted, FBG, HDL-C, and both diastolic and systolic blood pressure in the current study seemed to be influenced by a decrease in log total dietary fiber consumption. As in most proven hypotheses, dietary fiber is associated with increased HDL-C through the direct mechanism that increases bile and cholesterol fecal excretion, which subsequently stimulates the hepatic uptake to reduce LDL-C concentration from plasma [70,71]. The results derived from this study are in line with a cohort study in that a diet rich in fiber could play a significant role in the management and prevention of metabolic syndrome risk factors, including high blood pressure and an abnormal lipid profile [72]. It is also important to note that the prevalence of MetS obtained in the current research is similar to the MetS prevalence shown in black males (11%) and females (21%) participating in the study by Motala et al. [15]. While Motala et al. [15] did not investigate the effect of diet on the MetS risk factors it may be possible that the outcomes they obtained were influenced by insufficient dietary fiber consumption by their participants. After all, there is substantiated international evidence to suggest that the MetS risk factors, such as diabetes, cardiovascular, renal disease, and stroke are influenced by dietary intake [27,28,29,30,67]. Further evidence is needed in order to find which source of nutrients is more beneficial to the MetS milieu.

The strengths of the present study include the large community-based sample of both males and females, and the adjustments for potential confounders, especially total energy intake. Epidemiologic studies of diet encourage the use of total energy intake as a common confounder of diet–disease associations [73,74]. Therefore, energy intake was added as one of the confounders in the current study. However, we acknowledge that this study precludes any causal inferences; therefore, more longitudinal and experimental studies are needed before any firm conclusions can be drawn regarding the influence of different aspects of dietary fiber on the prevention of MetS.

This study may be of public health relevance, providing a potential link between dietary fiber consumption with other risk factors of metabolic syndrome. An emphasis on the consumption of food high in fiber might be a safe, effective, and low-cost approach to reducing risk factors of metabolic syndrome [75]. This could be of specific advantage for overweight and obese subjects at risk of developing MetS. Further studies are needed to assess whether the current findings can be generalized to other cohorts.

There are several limitations in this study. This study used the cross-sectional data to identify the association of dietary fiber intake with the metabolic syndrome. No specific dietary recommendations have been advocated by health agencies for the treatment of the metabolic syndrome. Therefore, in terms of implementing dietary change, emphases should be place on increasing dietary intake of foods high in fiber. Given that the metabolic syndrome is an identifiable and potentially modifiable risk state for both type 2 diabetes and cardiovascular disease, increasing dietary fiber intake may reduce the potential untoward effects of increasing risk factors of MetS [76]. Future studies using longitudinal data are required to ascertain which aspects of nutrition are linked to the prevention of metabolic syndrome. This study also acknowledges the limitation of analyzing a single dietary component on health outcomes. Physical activity was not measured in the current study participants; nonetheless, further studies are needed to include this confounder as it could strengthen the findings [75]. Moreover, given the observational nature of the present study, we cannot infer that the observed associations are causal. The participants in the present study were all from rural areas, which may limit the generalizability of our results to urban areas. Although, face-to-face 24-h dietary recall interviews administered by trained interviewers on one weekday and on one weekend day decreased the underestimation possibility. Finally, 24-h dietary recall data for a two-day period is not adequate because dietary habits typically differ each day from those on other weekdays. Thus, our 24-h dietary recall data might have underestimated some nutrients as well as energy intake.

## 5. Conclusions

The current study may be of public health relevance, providing a potential link between dietary fiber intake and FBG, HDL-C, and both systolic and diastolic blood pressure. Therefore, this observational data encourages public health policy measures to increase the consumption of dietary fiber in rural communities in order to lower the burden of MetS risk factors.

## Figures and Tables

**Table 1:** Descriptive statistics for total fiber, insoluble fiber, and soluble fiber intake amongst Ellisras adults aged 18–30 years.

	18–24 Years						25–30 Years						18–30 Years					
	Total Fiber (g)		Insoluble Fiber (g)		Soluble Fiber (g)		Total Fiber (g)		Insoluble Fiber (g)		Soluble Fiber (g)		Total Fiber (g)		Insoluble Fiber (g)		Soluble Fiber (g)	
	Males	Females	Males	Females	Males	Females	Males	Females	Males	Females	Males	Females	Males	Females	Males	Females	Males	Females
Median [IQR]	5.3 [0.0–32.1]	6.0 [0.0–32.7]	0.0 [0.0–6.7]	0.0 [0.0–10.9]	0.0 [0.0–7.3]	0.0 [0.0–32.7]	3.6 [0.0–43.9]	4.5 [0.0–48.9]	0.0 [0.0–12.0]	0.0 [0.0–18.0]	0.0 [0.0–4.8]	0.0 [0.0–14.9]	4.3 [0.0–43.9]	5.1 [0.0–48.9]	0.0 [0.0–12.0]	0.0 [0.0–18.0]	0.0 [0.0–7.3]	0.0 [0.0–14.9]
Total	5.3 [0.0–32.7]		0.0 [0.0–10.9]		0.0 [0.0–12.9]		4.0 [0.0–48.9]		0.0 [0.0–18.0]		0.0 [0.0–14.9]		4.6 [0.0–48.9]		0.0 [0.0–18. 0]		0.0 [0.0–15.0]	
SD Total	7.1 6.0 6.7		1.3 1.8 1.6		1.0 1.6 1.4		6.9 8.0 7.5		1.5 2.1 0.8		0.9 1.6 1.3		7.1 7.4 7.2		1.4 2.0 1.7		1.0 1.6 1.3	

IQR = Interquartile range: SD = standard deviation.

**Table 2:** Mean values of the metabolic syndrome (MetS) risk factors in young adults in Ellisras, South Africa.

	18–24 Years				25–30 Years				18–30 Years			
	Males (*n* = 122)	Female (*n* = 117)	Total (*n* = 239)	*p*-Value	Males (*n* = 182)	Female (*n* = 206)	Total (*n* = 388)	*p*-Value	Males (*n* = 304)	Females (*n* = 323)	Total (*n* = 627)	*p*-Value
Waist circumference (cm)	73.35 ± 8.80	78.11 ± 13.23	75.68 ± 11.42	0.001	76.23 ± 9.79	84.51 ± 14.49	80.63 ± 13.16	<0.001	75.07 ± 9.50	82.20 ± 14.36	78.74 ± 12.74	<0.001
HDL-C (mmol/L)	1.19 ± 0.33	1.10 ± 0.28	1.15 ± 0.312	0.027	1.21 ± 0.40	1.09 ± 0.31	1.14 ± 0.36	0.001	1.20 ± 0.37	1.09 ± 0.30	1.15 ± 0.34	<0.001
Fasting blood glucose (mmol/L)	5.51 ± 0.90	5.54 ± 0.95	5.52 ± 0.93	0.806	5.39 ± 0.86	5.62 ± 1.83	5.51 ± 1.46	0.121	5.44 ± 0.88	5.59 ± 1.56	5.51 ± 1.28	0.136
Total cholesterol (mmol/L)	3.98 ± 0.90	4.09 ± 1.01	4.03 ± 0.96	0.349	4.07 ± 0.95	4.36 ± 1.16	4.22 ± 1.08	0.007	4.03 ± 0.93	4.26 ± 1.11	4.15 ± 1.03	0.005
Triglycerides (mmol/L)	0.98 ± 0.60	0.85 ± 0.45	0.91 ± 0.54	0.067	1.11 ± 0.66	1.02 ± 0.53	1.06 ± 0.60	0.145	1.05 ± 0.64	0.96 ± 0.51	1.00 ± 0.58	0.035
Systolic blood pressure (mmHg)	123.25 ± 12.24	112.89 ± 10.26	118.18 ± 12.42	<0.001	127.66 ± 12.46	114.80 ± 11.11	120.83 ± 13.35	<0.001	125.89 ± 12.48	114.11 ± 10.83	119.82 ± 13.06	<0.001
Diastolic blood pressure (mmHg)	68.87 ± 9.51	68.74 ± 9.72	68.81 ± 9.59	0.916	73.09 ± 10.27	69.23 ± 9.13	71.04 ± 9.86	<0.001	71.39 ± 10.17	69.05 ± 9.33	70.19 ± 9.81	0.003

HDL-C = high-density lipoprotein cholesterol.

**Table 3:** The prevalence of dietary fiber consumption and components of metabolic syndrome in young adults in Ellisras, South Africa.

	18–24 Years				25–30 Years				18–30 Years			
	Males (*n* = 122)	Female (*n* = 117)	Total (*n* = 239)	*p*-Value	Males (*n* = 182)	Female (*n* = 206)	Total (*n* = 388)	*p*-Value	Males (*n* = 304)	Females (*n* = 323)	Total (*n* = 627)	*p*-Value
Metabolic syndrome	9.8 (12)	30.8 (36)	20.1 (48)	<0.001	7.7 (14)	40.3 (83)	25.0 (97)	<0.001	8.6 (26)	36.8 (119)	23.1 (145)	<0.001
Total fiber Male < 38 g Female < 25 g	95.9 (117)	100 (117)	97.9 (234)	0.027	96.7 (176)	99.0 (204)	97.9 (380)	0.108	96.4 (293)	99.4 (321)	97.9 (614)	0.008
Insoluble fiber < 10 g	100.0 (122)	99.1 (116)	99.6 (238)	0.308	99.5 (181)	99.5 (205)	99.5 (386)	0.930	99.7 (303)	99.4 (321)	99.5 (624)	0.599
Soluble fiber < 2.5 g	100.0 (122)	99.1 (116)	99.6 (238)	0.308	100.0 (182)	99.5 (205)	99.7 (387)	0.348	100.0 (304)	99.4 (321)	99.7 (625)	0.170
Elevated waist circumference Male > 94 cm Female > 80 cm	4.9 (6)	39.3 (46)	21.8 (52)	<0.001	4.4 (8)	60.2 (124)	34.0 (132)	<0.001	4.6 (14)	52.6 (170)	29.3 (184)	<0.001
Low HDL-C Male < 1.0 mmol/L Female < 1.3 mmol/L	29.5 (36)	77.8 (91)	53.1 (127)	<0.001	30.2 (55)	79.1 (163)	56.2 (218)	<0.001	29.9 (91)	78.6 (254)	55.0 (345)	<0.001
Elevated fasting blood glucose >5.6 mmol/L	45.1 (55)	47.9 (56)	46.6 (111)	0.711	42.9 (78)	47.6 (98)	45.4 (176)	0.353	43.8 (133)	47.7 (154)	45.8 (287)	0.343
High total cholesterol >5.1 mmol/L	10.7 (13)	17.9 (21)	14.2 (34)	0.108	11.5 (21)	24.8 (51)	18.6 (72)	0.001	11.2 (34)	22.3 (72)	16.9 (106)	<0.001
High triglycerides >1.7 mmol/L	8.2 (10)	7.7 (9)	7.9 (19)	0.888	12.1 (22)	9.7 (20)	10.8 (42)	0.453	10.5 (32)	9.0 (29)	9.7 (61)	0.514
High systolic blood pressure >130 mmHg	17.2 (21)	0.9 (1)	9.2 (22)	<0.001	24.7 (45)	4.9 (10)	14.2 (55)	<0.001	39.2 (100)	7.7 (25)	19.9 (125)	<0.001
High diastolic blood pressure >85 mmHg	6.6 (8)	6.8 (8)	6.7 (16)	0.931	10.4 (19)	3.9 (8)	7.0 (27)	0.011	8.9 (27)	5.0 (16)	6.9 (43)	0.052

HDL-C = high-density lipoprotein cholesterol.

**Table 4:** Linear regression analysis to show the relationship between log dietary fiber consumption and different metabolic syndrome risk factors in young adults in Ellisras.

	Log Total Fiber							
	Adjusted for Age and Gender				Adjusted for Age, Gender, and Energy			
	β	SE (Beta)	*p* Value	95% CI	β	SE (Beta)	*p* Value	95% CI
Waist circumference (cm)	0.000	0.001	0.899	(−0.003; 0.003)	0.001	0.001	0.788	(−0.002; 0.003)
HDL-C (mmol/L)	−0.028	0.052	0.592	(−0.128; 0.074)	−0.085	0.044	0.051	(−0.173; 0.002)
Fasting blood glucose (mmol/L)	−0.028	0.014	0.039	(−0.054; −0.001)	−0.019	0.012	0.044	(−0.042; 0.003)
Total cholesterol (mmol/L)	−0.007	0.017	0.687	(−0.040; 0.036)	−0.008	0.015	0.593	(−0.036; 0.021)
Triglycerides (mmol/L)	0.007	0.030	0.814	(−0.053; 0.067)	0.008	0.026	0.754	(−0.043; 0.59)
Systolic blood pressure (mmHg)	−0.003	0.001	0.033	(−0.006; 0.000)	−0.002	0.001	0.045	(−0.050; 0.002)
Diastolic blood pressure (mmHg)	−0.002	0.002	0.323	(−0.005; 0.002)	−0001	0.002	0.440	(−0.004; 0.002)
	**log insoluble fiber**							
Waist circumference (cm)	0.001	0.001	0.512	(−0.001; 0.002)	0.001	0.001	0.394	(−0.001; 0.002)
HDL-C (mmol/L)	−0.038	0.031	0.224	(−0.099; 0.023)	−0.055	0.030	0.071	(−0.114; 0.005)
Fasting blood glucose (mmol/L)	−0.013	0.008	0.103	(−0.019; 0.002)	−0.011	0.008	0.170	(−0.027; 0.005)
Total cholesterol (mmol/L)	−0.002	0.010	0.867	(−0.022; 0.018)	−0.002	0.010	0.840	(−0.021; 0.017)
Triglycerides (mmol/L)	0.005	0.018	0.765	(−0.031; 0.042)	0.006	0.018	0.745	(−0.029; 0.041)
Systolic blood pressure (mmHg)	−0.002	0.001	0.039	(−0.004; 0.000)	−0.002	0.001	0.061	(−0.003; 0.000)
Diastolic blood pressure (mmHg)	−0.001	0.001	0.317	(−0.003; 0.001)	−0.001	0.001	0.383	(−0.003; 0.001)
	**log soluble fiber**							
Waist circumference (cm)	0.001	0.001	0.465	(−0.001; 0.002)	0.001	0.001	0.353	(−0.001; 0.002)
HDL-C (mmol/L)	−0.035	0.026	0.179	(−0.087; 0.016)	−0.049	0.026	0.050	(−0.099; 0.001)
Fasting blood glucose (mmol/L)	−0.001	0.007	0.100	(−0.025; 0.002)	−0.009	0.007	0.166	(−0.022; 0.004)
Total cholesterol (mmol/L)	−0.004	0.009	0.676	(−0.020; 0.013)	−0.004	0.008	0.646	(−0.020; 0.031)
Triglycerides (mmol/L)	0.010	0.015	0.509	(−0.020; 0.041)	0.010	0.015	0.486	(−0.019; 0.040)
Systolic blood pressure (mmHg)	−0.002	0.001	0.018	(−0.003; 0.000)	−0.002	0.001	0.029	(−0.003; 0.000)
Diastolic blood pressure (mmHg)	−0.001	0.001	0.197	(−0.003; 0.001)	−0.001	−0.001	0.242	(−0.003; 0.001)

HDL-C = high-density lipoprotein cholesterol, CI = coefficient interval, SE = standard error.
